# Correlates of Presence of Feeding Difficulties in Children with Autism Spectrum Disorder and Other Developmental Conditions

**DOI:** 10.3390/nu18010010

**Published:** 2025-12-19

**Authors:** Tammy S. H. Lim, Pravin Anand, Ying Qi Kang, Jennifer S. H. Kiing, Mae Yue Tan, Shang Chee Chong, Liang Shen, Kalyani V. Mulay, Ramkumar Aishworiya

**Affiliations:** 1 Child Development Unit, Khoo Teck Puat-National University Children’s Medical Institute, National University Health System, Singapore 119074, Singapore; tammy_sh_lim@nuhs.edu.sg (T.S.H.L.); anand_pravin@nuhs.edu.sg (P.A.);; 2 Department of Paediatrics, Yong Loo Lin School of Medicine, National University of Singapore, Singapore 119228, Singapore; 3 Biostatistics Unit, Yong Loo Lin School of Medicine, National University of Singapore, Singapore 117597, Singapore

**Keywords:** feeding difficulties, developmental concerns, autism

## Abstract

**Background/Objectives**: Feeding difficulties are more common in children with autism spectrum disorder (ASD) or other developmental conditions and are associated with nutritional risk and caregiver stress. However, they may be overlooked as growth tends to be preserved. We aimed to identify clinical and behavioral features associated with feeding difficulties among children with developmental conditions. **Methods**: This cross-sectional study included caregiver–child dyads, with children aged 1–7 years with ASD and other developmental conditions. Caregivers completed the Repetitive Behavior Questionnaire, Second Edition (RBQ-2) to assess child restricted and repetitive behaviors (RRBs) and the Behavioral Pediatrics Feeding Assessment Scale (BPFAS) to assess feeding difficulties. Demographics, anthropometric measures and cognitive and adaptive scores were retrieved from medical records. **Results**: Of the 132 participants (mean age 41.8 months, range 15–67; 74.2% male) included, majority had normal weight (87.7%) and height (89.2%) *z* scores. Among participants, 54.5% had autism, 26.5% language delay and 18.9% other developmental diagnoses. Over half (53.0%) had elevated BPFAS scores. Children not enrolled in school showed significantly more feeding difficulties compared to those who were enrolled (32.6% vs. 16.7%, *p* < 0.05). The RBQ-2 total score positively correlated with the BPFAS total frequency score (*r* = 0.33, *p* = 0.01) after adjusting for gender, age and developmental diagnosis. **Conclusions**: Feeding difficulties were common in this sample. Higher RRBs and absence of formal schooling were associated with higher rates of feeding difficulties. Longitudinal studies are needed to ascertain the role of RRBs and school enrollment as clinical indicators associated with feeding difficulties.

## 1. Introduction

Feeding difficulties are common in children with autism spectrum disorder (used here analogously with autism) and other developmental conditions with consequent nutritional risks and adverse health consequences. Compared to typically developing children, children with developmental delays or related conditions have higher rates of feeding difficulties [1]. Conversely, those who experience feeding difficulties are also more likely to have developmental delays [2], with one study showing the incidence of developmental and behavioral difficulties to be 20% in a large cohort of children with feeding difficulties [3]. Specifically, amongst autistic children, feeding difficulties are described in up to 75% [4], including in Singapore, where this has been reported in more than a quarter of autistic children [5]. Further, a study from a local multidisciplinary feeding clinic showed that, amongst children presenting with “picky eating”, the majority engage in a highly selective diet (acceptance of fewer than 15 food items), and more than half of these children have a diagnosis of autism [6].

In spite of clinician awareness of the prevalence of feeding difficulties in this population of children, problematic feeding issues may not be fully addressed. There may be limited time in the clinic consult as the clinician evaluates the top three concerns of parents of children with autism spectrum disorder, i.e., social communication difficulties, repetitive behaviors, and restricted interests and behavioral challenges [7]. Further, physicians are not cued into the presence of feeding difficulties, as children often maintain their weight and height; thus, these are not necessarily reliable surrogate markers to suggest the presence of feeding difficulties. For instance, in a local cohort of autistic children with highly selective diets, the majority had normal weight and height, despite many having diets deficient in macronutrients or micronutrients (e.g., iron, calcium) [8]. This finding is consistent in other cohorts and is thought to be related to the preference for high-calorie low-nutrient processed foods, which are more consistent in sensory properties, such as appearance, taste and texture [9,10]. Feeding difficulties affect family mealtimes and contribute to caregiver stress [11]. Therefore, there is a need to identify other clinical correlates that may be indicative of the presence of or association with a higher likelihood of feeding difficulties.

While restricted and repetitive behaviors (RRBs) are best known in autism spectrum disorder, where RRBs are one of the two core symptoms, they are also common in non-autistic young children [12]. RRBs have also been described in attention deficit hyperactivity disorder, psychotic disorders, anxiety and obsessive–compulsive disorder, as well as genetic syndromes like Prader–Willi, Williams syndrome and Fragile X syndrome [12,13]. In a 2022 systematic review examining the correlates of feeding difficulties in autistic children, there was an equivocal association between RRBs and feeding difficulties [14]. While children with developmental delays experience RRBs and feeding difficulties, to date there are no published studies exploring the relationship between RRBs and feeding difficulties in this population. During time-scarce healthcare visits, RRBs may be clinically more apparent or pressing concerns of parents, compared to feeding difficulties, which may not be brought up unless the clinician specifically takes a dietary history. The primary objective of this study is to identify clinical and behavioral features (including RRBs) associated with higher presence of feeding difficulties among children with developmental conditions. We hypothesized that a greater number of RRBs will be associated with more severe feeding difficulties and that these feeding difficulties will be more common among children with autism spectrum disorder as compared to other developmental conditions.

## 2. Materials and Methods

### 2.1. Study Setting and Participants

Data presented in this manuscript was obtained from a cross-sectional study in which parent/caregiver–child dyads were recruited over a 2-year period between September 2019 and October 2021 in a Developmental–Behavioral Pediatrics (DBP) clinic situated within a tertiary academic center in Singapore. The Child Development Unit at National University Hospital (NUH) is one of two national DBP centers in Singapore, offering diagnostic and intervention services for children from birth until 7 years of age, supporting approximately 13,000 outpatient visits annually. Our center evaluates children referred by their primary care physicians for any developmental, behavioral, learning, or emotional concerns. The multidisciplinary team at the center provides diagnostic evaluations, developmental assessments, diagnosis-specific intervention and referral services for community partners and for its patients.

Inclusion criteria for this study included the following:

1. Child age 1–7 years old,
2. Child with developmental conditions (including autism spectrum disorder, isolated language delay and global developmental delay as per Diagnostic and Statistical Manual of Mental Disorders, Fifth Edition (DSM-5) criteria), and
3. Caregiver proficiency in English.

English language literacy rates are high at >80% within the country/city where this study was based in. Further, this is the main language of instruction in local schools and is also the most frequently spoken language at home in Singapore [15]. Hence, the inclusion criteria of caregiver proficiency in the English language would be considered representative of the population.

This cohort did not include any children with chronic medical conditions, which could affect their feeding. As this was a cross-sectional study to explore associations, sample size calculation was not conducted.

### 2.2. Ethics Approval

Institutional review board approval was obtained prior to the commencement of this study (National Healthcare Group Domain Specific Review Board [NHG DSRB], Reference Number: 2019/00132). Consent was obtained from either parents or caregivers of the children who were recruited in this study.

### 2.3. Procedure

Following informed consent, caregivers completed the following questionnaires under study measures listed below. In addition, clinical data was also obtained from medical records for each child as detailed below.

#### 2.3.1. Caregiver-Completed Study Measures

##### Repetitive Behavior Questionnaire, Second Edition (RBQ-2)

Repetitive Behavior Questionnaire-2nd edition (RBQ-2) is a caregiver-completed 20-item questionnaire designed to measure RRBs in children [12]. It was selected as it has good internal consistency and validity. In addition, we selected this tool as it is used in children as young as 15 months of age, which was appropriate for our clinical cohort, while other tools that measure RRBs, for example, the Repetitive Behaviors Scale, Revised, were typically used for older children. The mean total score was computed by adding the responses for each item (score range of 1 to 3), divided by the total number of items. Subscale scores (motor/sensory behaviors (MS) and rigidity/routines/preoccupation with restricted interests (RRPRI)) were similarly derived. A higher score indicates the presence of more frequent repetitive behaviors, on a scale of 1 to 3. The mean total score and the RRPRI subscale score were calculated with and without item 19 (“Does your child insist on eating the same foods, or a very small range of foods, at every meal”) to determine if feeding difficulties will skew the scores.

##### Behavioral Pediatrics Feeding Assessment Scale (BPFAS)

The BPFAS [16] is a validated tool used to measure caregiver-reported childhood mealtime behaviors, and in this study it is used to determine the presence of feeding difficulties. We chose this tool as it has high sensitivity and specificity and good reliability in detecting feeding problems in children [17]. In addition, it has been used in children on the autism spectrum and has demonstrated good psychometric properties in this cohort [18]. The BPFAS comprises 35 questions. There are four separate sections with scores: child frequency score (CFS), parent frequency score (PFS), child problem score (CPS) and parent problem score (PPS); CFS and PFS are computed to derive the total frequency score (TFS). Scores higher than specific cut-offs in any section suggest clinically significant feeding difficulties: 84 for total frequency score (TFS), 61 for child frequency score (CFS), 20 for parent frequency score (PFS), 6 for child problem score (CPS) and 2 for parent problem score (PPS), while higher TFSs suggest more severe feeding difficulties [19].

#### 2.3.2. Demographics Questionnaire

Demographic information on caregiver and child was collected using a standardized questionnaire, completed by caregivers. Information collected included age and highest educational qualification of caregiver, age, gender, school enrollment status, and presence of siblings for the child.

#### 2.3.3. Growth Data of Child

Growth parameters obtained from electronic medical records included weight and height of the child, which are routinely measured at every clinic visit in our center. Weight was measured to the nearest 0.1 kg, while height was measured to the nearest 1 mm. Weight and height *z* scores were calculated based on the World Health Organization Growth Standards. For weight *z* scores, less than −2 is defined as underweight, −2 to 2 normal weight and above 2 as overweight. For height *z* scores, less than −2 is considered stunted growth, while −2 and above is regarded as normal.

#### 2.3.4. Clinical and Development-Related Data of Child

An electronic medical record review was conducted to obtain the following information on the child, as of either the actual date or within the closest time frame of study participation: developmental diagnosis (based on DSM-5 criteria), cognition, adaptive skills and Social Responsiveness Scale results. Child cognition was assessed as part of routine clinical care for each child using standardized assessments, according to the age of the child; results of these assessments were abstracted from electronic medical records for each child. These included the Mullen Scales of Early Learning (MSEL) and the Kaufman Brief Intelligence Test, Second Edition (KBIT-2). Both these standardized measures have good validity when compared with other tests to assess a child’s cognition [20,21]. The MSEL measures development in children from birth to 68 months with scores in five subscales: gross motor, visual reception, fine motor, receptive language and expressive language [22]. Scores for each subscale are generated based on a standard mean score of 50, with a standard deviation (SD) of 10. The scores from the four cognitive subscales (visual reception, fine motor, receptive language and expressive language) can be computed to yield an Early Learning Composite (ELC) score, with a mean T score of 100 (SD 15), as an approximation of a child’s cognition. KBIT-2 is a brief test of verbal and nonverbal intelligence that comprises three subtests. It produces three scores, verbal IQ (VIQ) score, nonverbal IQ (NVIQ) score and an IQ composite score, and is used in children older than 68 months of age [23].

Vineland Adaptive Behavior Scales, Second Edition (VABS-II) measures the adaptive skills of an individual (from birth to 90 years of age). This was performed by trained psychologists in our center as part of routine clinical care for study participants. From the VABS-II, the Adaptive Behavior Composite (ABC), which is a standardized score of the overall level of adaptive skills, is derived; this information was abstracted from electronic medical records where available and was used for study analysis.

Social Responsiveness Scale, 2nd edition (SRS-2) is a tool used to measure difficulties related to autism traits, specifically social communication and restricted behaviors. The preschool rating form (for ages 2.5–4 years) and the school-age form (for ages 4–18 years) were used. These were completed by the parent/caregiver of children with suspected autism or social communication difficulties, as part of routine clinical care. The SRS-2 generates a total T score with a mean (SD) of 50 (10) following completion; this information was abstracted from electronic medical records and was used for analysis in this study.

### 2.4. Statistical Analysis

All analyses were performed using IBM SPSS Statistics for Windows, Version 28.0 (Armonk, NY, USA: IBM Corp). Statistical significance was set at *p* < 0.05 (2-sided). Internal consistency of the BPFAS and RBQ-2 were assessed using Cronbach’s alpha (α). Descriptive statistics are presented as mean (SD) for normally distributed numerical data and *n* (%) for categorical variables. Comparative analysis using chi-square tests and correlational analysis using Spearman’s correlation was performed. Spearman’s rho correlation coefficient of 0.20–0.39 describes a mild correlation, 0.40–0.69 moderate, while 0.70–1.0 describes a strong correlation. Chi-square tests were used to study the association between potential risk factors and feeding difficulties, and Spearman’s correlation was used to evaluate the association between RBQ-2 and BPFAS scores. Logistic regression was used to adjust for confounders in statistically significant relationships between demographic and clinical variables and feeding difficulties.

## 3. Results

### 3.1. Descriptive Data on Caregiver and Child Characteristics

A total of 132 caregiver–child dyads were recruited. The mean ± SD (range) age of the children was 41.8 ± 11.8 months (15–67), with the majority (74.2%) being male (Table 1). Most children demonstrated normal growth rates: 87.7% (*n* = 130) had weight *z* scores from −2 to +2, while 89.2% (*n* = 130) had height *z* scores of −2 and above. Half (54.5%) the cohort was on the autism spectrum, while 26.5% had speech and language delay and 18.9% had other developmental diagnoses. The average cognitive score was in the borderline range at 78.2 ± 26.0. The parents in this group were relatively highly educated with more than half the parents having tertiary education.

### 3.2. BPFAS Results and Clinical/Demographic Factors Associated with Higher BPFAS Scores

Half of the total cohort (53.0%, n = 53) reported clinically significant feeding difficulties on the BPFAS (Table 2). Cronbach’s alpha score for the BPFAS is as follows: child frequency 0.83, parent frequency 0.91, child problem 0.78, parent problem 0.89, indicating good internal consistency. Weight and height *z* scores were not statistically significantly associated with the BPFAS TFS, yielding *p* = 0.42 and *p* = 0.56, respectively.

Univariate analysis showed that children who were not enrolled in school were more likely to have significant feeding difficulties (reported on BPFAS); 32.6% vs. 16.7% (*p* = 0.04) (Table 3). After adjusting for age and cognition, which showed significant association with school enrollment, children who were not enrolled in school still showed a trend towards being more likely to have feeding difficulties, compared to those who were enrolled in school (adjusted odds ratio 2.41 (95% CI 0.92–6.00, *p* = 0.06). We did not adjust for developmental diagnosis (*p* = 0.82), or other variables in Table 3, as they were not confounders. There was a trend towards those with fine motor delay and those with a higher degree of parent-reported child social communication difficulties on SRS of being more likely to have feeding difficulties on the BPFAS: 31.8% vs. 17.4% (*p* = 0.06) and 32.6% vs. 17.5% (*p* = 0.08), respectively. Other factors (child gender, developmental diagnosis, cognition, adaptive skills, presence of siblings, enrolment in school, other forms of developmental delay, parent educational level) were not associated with higher likelihood of having feeding difficulties on the BPFAS.

Logistic regression including the above family demographic and clinical variables (SRS score, fine motor delay) with a *p* value of < 0.1 in univariate analysis did not identify any factors as significantly associated with the presence of feeding difficulties.

### 3.3. RBQ-2 Results

Scores from the RBQ-2 are presented in Table 4. The mean ± SD (range) RBQ-2 total score was 1.54 ± 0.31 (1–2.35). When the question on feeding difficulties within the RBQ-2 (“Does your child insist on eating the same foods or a very small range of food at every meal?”) was excluded, the RBQ-2 total score remained very similar: 1.54 ± 0.32 (1–2.37). The autism group had a slightly higher score at 1.60 ± 0.30, compared to the non-autism group 1.47 ± 0.31; *p* = 0.01. Cronbach’s α was 0.85.

Spearman’s correlation was conducted to evaluate the relationship between RBQ scores and severity of feeding difficulties reported on the BPFAS TFS (Table 5). The RBQ-2 total score positively correlated with mild strength with the BPFAS TFS (*r* = 0.33, *p* = 0.01). Specifically, this was seen with mild strength in both the RBQ-2 MS and RRPRI subscales, respectively; (*r* = 0.24, *p* = 0.01), (r = 0.37, *p* = 0.01). This relationship remained true even after exclusion of the question on feeding difficulties (as noted above). After adjusting for gender, age and developmental diagnosis of the child, the relationship between RBQ-2 and BPFAS TFS remains of similar strength.

## 4. Discussion

In our study of children with autism spectrum disorder, speech and language delay and other developmental concerns, more than half have parent-reported feeding difficulties on the BPFAS. Our other key results include an association between the presence of RRBs and feeding difficulties within this sample.

The incidence of feeding difficulties in our sample is higher than in typically developing children [24], consistent with what has been reported in the literature [25]. In this cohort, the BPFAS subscale score with the highest proportion of parents exceeding cut-offs was the parent frequency score. Examples of the questions in this section included the following: parental feelings of frustration and anxiety when feeding the child, parental confidence in feeding the child, disagreement with family members on feeding, strategies used by parents (coaxing, threatening, making alternative foods), and feeling angry with the child. This finding suggests that parents of children with developmental delays or disorders can experience significant negative emotions or stress surrounding mealtimes and may employ maladaptive strategies at mealtimes [26,27,28]. This group of parents may benefit from tailored caregiver training to improve their confidence and competence in managing their child’s feeding difficulties. There is literature available to suggest that parents who are experiencing poorer confidence in managing their child’s feeding difficulties demonstrate an improvement in their confidence following training by a trained professional to deliver caregiver-mediated feeding strategies at home [29].

Growth was preserved in the majority of patients in this cohort, despite having a high prevalence of feeding difficulties. This is consistent with what is currently reported in the literature [8]. Hence, having poor growth is likely a late sign of severe and persistent feeding difficulties, and an earlier indicator of such issues is needed to prompt clinicians to detect and manage them before complications develop. Our study found two correlates that are associated with increased likelihood of feeding difficulties: increased RRBs and the lack of school enrolment. This was consistent with findings from a Malaysian cohort study of autistic children aged between 1 and 7 years old [30]. RRBs are one of the core symptoms of autism and can significantly influence feeding patterns and mealtime behavior. These behaviors can arise from underlying insistence on sameness, repetitive motor movements, restricted interests and rigid adherence to routines. When it comes to feeding, RRBs often manifest as strong preferences for specific foods, brands, or meal presentations and resistance to any change in mealtime routines or food appearance. Children with RRBs may insist on eating the same foods prepared and presented in the same way each day, displaying distress or refusal if even small changes occur—such as differences in food color, texture, or packaging—making dietary expansion and the introduction of new foods particularly challenging. Whilst our study methodology does not allow for conclusions on causality about the relationship between higher levels of RRBs and feeding difficulties, RRBs and feeding difficulties in general are not known to share a causal relationship in autistic children. Rather, feeding difficulties are known to be one of the manifestations of RRBs, as detailed in the DSM-5 criteria [31]. Having to “eat (the) same food every day” is listed as one of the examples under criteria B2 (“insistence on sameness, inflexible adherence to routines, or ritualized patterns of verbal or nonverbal behavior”) of the DSM-5 criteria for autism spectrum disorder. In the long run, highly selective eating patterns are known to result in nutritional deficiencies, with patients reported to develop conditions that are typically rare in the developed world, such as, vitamin A-related blindness or scurvy [32]. Strategies such as gradual exposure to new foods, structured mealtime routines and collaboration with occupational or behavioral therapists can help reduce rigidity and increase food acceptance. By addressing both the behavioral and sensory aspects of feeding within the context of children with RRBs, caregivers and professionals can create supportive, predictable and flexible mealtime environments that promote healthier eating habits and reduce mealtime stress. In non-autistic children with developmental needs, the relationship between RRBs and feeding difficulties is not so well understood and needs further research to better understand this association. There are currently no published studies that have evaluated this association. Nonetheless, when a clinician sees a child with a high level of RRBs, particularly RRPRI type, regardless of the developmental diagnosis, this should prompt a more detailed feeding history. Clinicians may need to ask if the child is eating from all food groups, since there is data to suggest that, especially amongst the autistic children, complete omission of specific food groups is common [8].

In addition, our study also found that children not enrolled in preschool had more significant feeding difficulties reported on BPFAS compared to those who were enrolled; 32.6% vs. 16.7% (*p* = 0.04). This relationship remained almost close to statistical significance (*p* = 0.06) even after adjusting for possible confounders. It is possible that repeating this study with a larger sample size might achieve a more statistically significant result and demonstrate that the lack of school enrollment is independently associated with feeding difficulties. This finding appears consistent with observations that typically developing children who attend preschool are less likely to have severe picky eating and are more likely to have a greater food repertoire [33]. This is because preschool environments encourage repeated exposure to unfamiliar foods, positive social experiences around mealtimes, and peer modeling, all of which help to facilitate acceptance of a wider range of food and reduce selective eating behaviors [34,35,36]. Although the existing literature has yet to establish direct evidence between preschool enrollment and reduction in feeding difficulties in children with developmental delays or disorders, the protective effects of preschool enrollment to feeding and overall development in general could apply to children with developmental delays or disorders and remains an important area for future research. Conversely, children who do not attend school may be at higher risk of experiencing feeding difficulties because they have less exposure to structured mealtime routines, peer modeling and diverse food environments, which are protective factors against feeding problems [33]. Preschool attendance provides regular opportunities for children to observe and imitate peers and adults eating a variety of foods, which can reduce food neophobia and picky eating habits [35,36]. Children who do not attend preschool may experience less social facilitation of eating, more parental accommodation of food preferences, and potentially less exposure to new foods, all of which are associated with increased risk of feeding difficulties and selective eating [34,36]. The home feeding environment, including parental feeding practices and mealtime structure, plays a critical role; less structured environments and greater child control over food choices are linked to higher rates of picky eating and feeding problems [36]. As our study had a cross-sectional design, we acknowledge that we are unable to conclude causality in the relationship between school enrolment and feeding difficulties. It is thus also plausible that parents are less likely to enroll their child in school if the child is already experiencing significant feeding difficulties, as parents may be concerned that the child would go hungry in school. Further longitudinal studies can examine this relationship in order to understand potential causality or bidirectionality. Nonetheless, based on our results that suggest an association in this area, for children who are experiencing feeding difficulties, clinicians should engage with parents to find out if the child is enrolled in school. Conversely, for those children who are not enrolled in school, clinicians may be prompted to find out if one of the reasons could be due to the presence of feeding difficulties and parental concerns related to this.

### Limitations

This study involved convenience sampling of parent/caregiver–child dyads attending a developmental–behavioral pediatric clinic, putting the sample at risk of self-selection bias as participants may be more likely to participate if their child had more RRBs or feeding difficulties. The study’s cross-sectional design does not allow for capturing longitudinal information on childhood feeding difficulties or RRBs, both of which may evolve over time, changing the relationship between these two factors. Study results thus depict associations and do not allow inference related to possible causality. Future research can be performed to evaluate long-term correlates of feeding difficulties in children with developmental needs to understand if and how they may change over time. In addition, this study compared parent-completed questionnaire-based measures of feeding difficulties and repetitive behaviors in children (BPFAS, RBQ-2), and these measures may not be entirely objective, thereby limiting reliability of measurements. Use of more objective/clinician-assessed measures of feeding difficulties and repetitive behaviors can enhance accuracy of derived measures in future studies.

## 5. Conclusions

While growth was preserved, presence of feeding-related difficulties was common in this sample of children with developmental conditions. Increased RRBs and lack of school enrollment were associated with higher rates of feeding difficulties in this group of children and should prompt earlier identification of these at-risk children. Children with higher levels of RRBs may have sensory preferences and insistence of sameness that result in a more selective eating pattern [37]. In such children, the clinician should be prompted to take a more detailed dietary history, specifically to find out if the child has selective eating and is omitting any food groups entirely. Clinicians should also routinely find out about school enrollment status in these children, since school enrollment (through the provision of structured mealtimes, peer modeling, food variety, etc.) appears protective against feeding difficulties. Eliciting information about RRBs and school enrollment can potentially facilitate identification of children for further evaluation and intervention regarding feeding difficulties.

## Figures and Tables

**Table 1:** Background characteristics of study participants (*n* = 132).

Characteristics	Value
Age (months)	41.8 ± 11.8 (15–67)
Gender	
Male	98 (74.2)
Female	34 (25.8)
Weight (*n* = 130)	*z* score −0.08 ± 1.30 (−3.12–5.30)
Underweight (*z* score < −2)	10 (7.7)
Normal weight (*z* score −2 to +2)	114 (87.7)
Overweight (*z* score > 2)	6 (4.6)
Height (*n* = 130)	*z* score −0.42 ± 1.36 (−5.00–2.90)
Stunted (*z* score < −2)	14 (10.8)
Normal height (*z* score −2 and above)	116 (89.2)
School enrollment	
Yes	84 (63.6)
No	46 (34.8)
Presence of siblings (*n* = 130)	
Yes	74 (56.1)
No	56 (42.4)
Father’s age	37.5 ± 6.8 (21–62)
Mother’s age	34.2 ± 5.0 (20–47)
Father’s highest educational qualification (*n* = 113)	
Primary school	3 (2.3)
Secondary school	13 (9.8)
Post-secondary school	23 (17.4)
Diploma	28 (21.2)
Graduate degree	35 (26.5)
Post-graduate	11 (8.3)
Mother’s highest educational qualification (*n* = 117)	
Primary school	1 (0.8)
Secondary school	19 (14.4)
Post-secondary school	17 (12.9)
Diploma	29 (22.0)
Graduate degree	38 (28.8)
Post-graduate	13 (9.8)
Developmental diagnosis	
Autism	72 (54.5)
Speech and language delay	35 (26.5)
Others *	25 (18.9)
Cognition ^‡^ (*n* = 130)	78.2 ± 26.0 (49–141)
Cognitive score < 70	55 (42.3)
Cognitive score ≥ 70	75 (57.7)
Adaptive skills ^§^ (*n* = 37)	
Adaptive score < 70	16 (43.2)
Adaptive score ≥ 70	21 (56.8)
Social Responsiveness Scale (*n* = 100)	
Parent questionnaire, total T score ≥ 60 ^~^	43 (43.0)

Data are presented as *n* (%) or mean ± SD (range). * Global developmental delay (12), articulation difficulties (3), behavior concern (7), motor delay (1), developmental surveillance (2). ^‡^ Based on the Mullen Scales of Early Learning (MSEL) or the Kaufman Brief Intelligence Test, 2nd ed. (KBIT-2); *n* = 130. ^§^ Based on the Vineland Adaptive Behavior Scales—3rd ed. (Vineland-3); *n* = 37. ^~^ Total T scores of 60 and above on the Social Responsiveness Scale—2nd ed. (SRS-2) indicates clinically significant social difficulties; *n* = 100.

**Table 2:** Results from the Behavioral Pediatrics Feeding Assessment Scale (BPFAS).

BPFAS Metrics	*n* = 132
Exceeded cut-off in any BPFAS subscale	70 (53.0)
Total Frequency Score (TFS)	71.7 ± 17.3 (39–124)
% with high TFS (i.e., >84)	29 (22.0)
Child Frequency Score (CFS)	51.1 ± 12.1 (29–88)
% with high CFS (i.e., >61)	24 (18.2)
Parent Frequency Score (PFS)	20.6 ± 6.3 (10–38)
% with high PFS (i.e., >20)	68 (51.5)
Child Problem Score (CPS)	3.2 ± 4.6 (0–20)
% with high CPS (i.e., >6)	24 (18.2)
Parent Problem Score (PPS)	1.4 ± 2.4 (0–10)
% with high PPS (i.e., >2)	27 (20.5)

Data are presented as *n* (%) or mean ± SD (range).

**Table 3:** Univariate analysis of factors associated with higher total frequency score (TFS) on the Behavioral Pediatrics Feeding Assessment Scale (BPFAS).

	***n*** **(%) with Significant TFS (>84)**	***p*** **Value**
**Child Factors**		
Gender		0.46
Male	20 (20.4)	
Female	9 (26.5)	
Enrollment in school		0.04 *
Yes	14 (16.7)	
No	15 (32.6)	
Presence of siblings		
Yes	16 (26.1)	0.78
No	11 (19.6)	
Developmental diagnosis		0.93
Autism	16 (22.2)	
Speech and language delay	7 (20.0)	
Others	6 (24.0)	
Cognition ^†^		0.11
Score < 70	16 (29.1)	
Score ≥ 70	13 (17.3)	
Adaptive skills ^‡^		
Score < 70	5 (31.3)	0.89
Score ≥ 70	7 (33.3)	
Developmental delay (on MSEL)		
Fine motor delay		
Present	14 (31.8)	0.06 **
Absent	15 (17.4)	
Receptive language delay		
Present	14 (28.6)	0.18
Absent	15 (18.5)	
Expressive language delay		
Present	15 (25.0)	0.50
Absent	14 (20.0)	
Social Responsiveness Scale score (rated by parent)		0.08 **
Within normal limits	10 (17.5)	
Elevated	14 (32.6)	
**Parent Factors**		
Father’s educational level		0.77
Below diploma level	7 (17.9)	
Diploma and above	15 (20.3)	
Mother’s educational level		0.36
Below diploma level	6 (16.2)	
Diploma and above	19 (23.8)	

^†^ Based on the Mullen Scales of Early Learning (MSEL) or the Kaufman Brief Intelligence Test, 2nd ed. (KBIT-2). ^‡^ Based on the Vineland Adaptive Behavior Scales—3rd ed. (Vineland-3). * *p* < 0.05. ** 0.05 ≥ *p* < 0.1.

**Table 4:** Results from Repetitive Behavior Questionnaire, Second Edition (RBQ-2).

RBQ-2 Metrics		*n* = 130 Mean ± SD (Range)
RBQ-2 total		1.54 ± 0.31 (1–2.35)
RBQ-MS ^‡^		1.53 ± 0.42 (1–2.78)
RBQ-2 RRPRI ^§^		1.48 ± 0.34 (1–2.63)
RBQ-2 total, excluding question on feeding difficulties *		1.54 ± 0.32 (1–2.37)
RBQ-2 RRPRI, excluding question on feeding difficulties *		1.48 ± 0.36 (1–2.57)
RBQ-2 scores in children with autism	RBQ-2 scores in children without autism	*p*
1.60 ± 0.30	1.47 ± 0.31	0.01

Data is presented as mean ± SD (range). ^‡^ RBQ-MS RBQ Motor/Sensory Behaviors. ^§^ RBQ-RRPRI, RBQ Rigidity/Routines/Preoccupation with Restricted Interests. Data is presented as mean ± SD (range). * Does your child insist on eating the same foods, or a very small range of foods, at every meal?

**Table 5:** Association between RBQ-2 and BPFAS total frequency scores.

	*r*	*r* (Excluding Question on Feeding Difficulties)	Adjusted *r*^
RBQ-2 total	0.33 **	0.30 **	0.32 **
RBQ-2 MS	0.24 **	0.24 *	0.24 **
RBQ-2 RRPRI	0.37 **	0.29 **	0.35 **

RBQ-2, Repetitive Behavior Questionnaire, Second Edition; RBQ-MS, RBQ Motor/Sensory Behaviors; RBQ-RRPRI, RBQ Rigidity/Routines/Preoccupation with Restricted Interests. *r*^, adjusted *r* for gender, age, and developmental diagnosis. *N* = 132 for *r*, *r*^. * *p* < 0.05; ** *p* < 0.01.

## Data Availability

The data presented in this study are available on request from the corresponding author due to privacy concerns.

## Abbreviations

The following abbreviations are used in this manuscript:

DSM-5	Diagnostic and Statistical Manual of Mental Disorders, Fifth Edition
RRBs	Restricted and repetitive behaviors
RBQ-2	Repetitive Behavior Questionnaire-2nd edition
RRPRI	Preoccupation with restricted interests
MS	Motor/sensory behaviors
BPFAS	Behavioral Pediatrics Feeding Assessment Scale
CFS	Child frequency score
PFS	Parent frequency score
CPS	Child problem score
PPS	Parent problem score
TFS	Total frequency score
MSEL	Mullen Scales of Early Learning
KBIT-2	Kaufman Brief Intelligence Test, Second Edition
ELC	Early Learning Composite
VIQ	Verbal IQ
NVIQ	Nonverbal IQ
VABS-II	Vineland Adaptive Behavior Scales, Second Edition
ABC	Adaptive Behavior Composite
SRS-2	Social Responsiveness Scale, 2nd edition
SD	Standard deviation
